# Supplementary material for: Impact of misclassified defective proviruses on HIV reservoir measurements
Source: Nat Commun. 2023 Jul 13;14:4186. doi: 10.1038/s41467-023-39837-z (PMC10345136; doi:10.1038/s41467-023-39837-z)
Supplement: Supplementary file 1 — Supplementary Information [file 41467_2023_39837_MOESM1_ESM.pdf]

Supplementary Information

Impact of misclassified defective proviruses on HIV reservoir measurements

Daniel B Reeves* [1], Christian Gaebler [2,3], Thiago Y Oliveira [2], Michael J Peluso [4], Joshua T Schiffer [1,5], Lillian B Cohn [1], Steven G Deeks [4], Michel C Nussenzweig [2,6]

[1] Vaccine and Infectious Disease Division, Fred Hutchinson Cancer Center, Seattle, WA, USA
[2] Laboratory of Molecular Immunology, The Rockefeller University, New York, NY, USA
[3] Laboratory of Translational Immunology of Viral Infections, Department of Infectious Diseases, Charité -Universitätsmedizin, Berlin, Germany
[4] Division of HIV, Infectious Diseases, and Global Medicine, Department of Medicine, UCSF, San Francisco, California, USA
[5] Department of Medicine, University of Washington, Seattle, WA, USA
[6] Howard Hughes Medical Institute, The Rockefeller University, New York, NY, USA
*Correspondence to dreeves@fredhutch.org

Supplementary Table 1. Summary of prior IPDA measurements of intact HIV proviral decay.

Publication	N	Intact reservoir half-life	Notes
Bruner et al. Nature (2019)[2]	13	44 mo (N=7), 100-300 mo (N=3), >700 mo (N=3)	Decays were estimated using 2 time points relative to study time (not time on ART).
Gandhi et al. JID (2020)[3]	44	median 84, 95% CI=[47-216] mo until year 7, 224 95% CI=[98-infinite] mo after year 7	study began after 7 years of ART, so study timepoints were at roughly 7, 11, and 13 years of ART (32 male, 12 female)
Antar et al. JCI (2020)[4]	8	mean 29, 95% CI=[20-52] mo	~2 time points per person. Log-linear mixed effects model, 1 PWH with upward trend in intact.
Falcinelli et al. JID (2020)[5]	29	median 16 mo (very rough, numerical values not reported in text)	~3 years of study time and ~4 time points per person. Log10 linear mixed effects model. Only report % change per year, so we used ln(2)/-ln(%)*12 to get half-life in mo and got % change by eye from fig2
Levy et al. Cell Rep Med (2021)[6]	20	52 mo (N=11), >120 mo (N=9)	~8 longitudinal samples per person, beginning Population nonlinear mixed effects model
White et al. PNAS (2021)[7]	15	mean 19, 95% CI=[8-44] mo	~14 longitudinal samples per person beginning at ART initiation (or reinitation after 6 mo pause). Population nonlinear mixed effects models and biphasic decays were tested (required for intact but not defective proviruses). First phase half-life ~13 days means stabilization to terminal half-life occurs within 3-4 mo post ART.
Peluso et al. JCI Insight (2021)[8]	81	48, [32-100] mo	~3 time points per person. >95% male, >70% White. Linear spline model with 1 knot (unclear if was log-linear but we assumed it was)

Abbreviations: mo, months

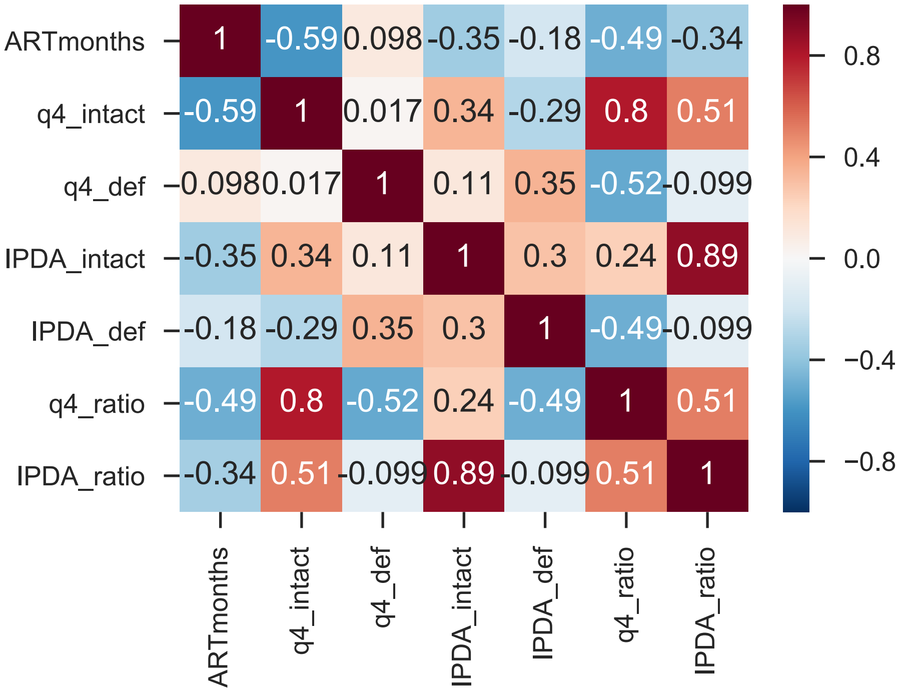

Supplementary Figure 1. Heatmap table of Spearman correlation coefficients across and between assays for both proviral categories and over all longitudinal time points.
Red indicates more positively correlated whereas blue indicates more negatively correlated. White indicates uncorrelated. q4_ and IPDA_ prefixes indicate Q4PCR and IPDA values, respectively. Correlations were calculated for all n=35 timepoints from 10 PWH.

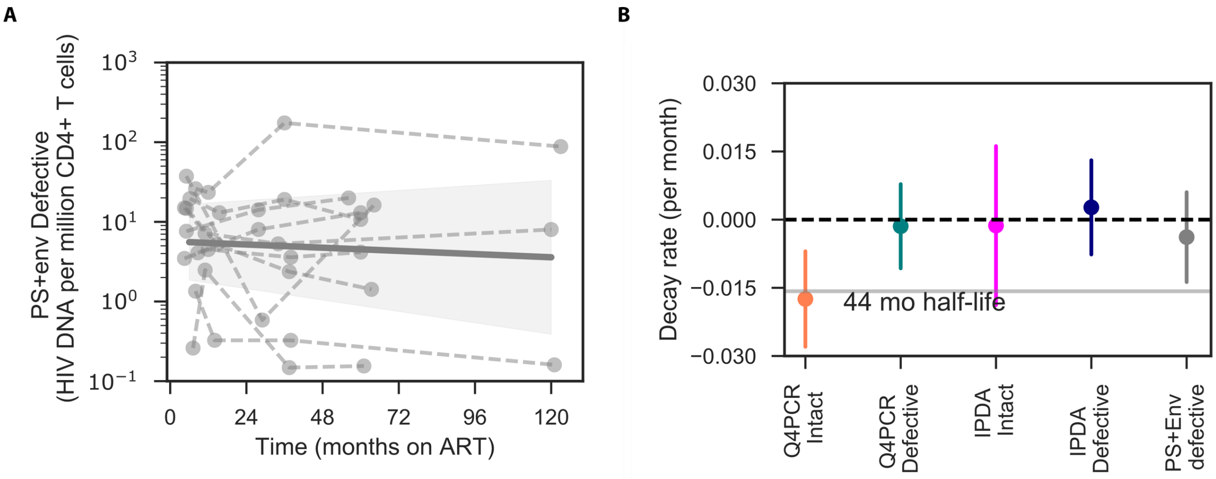

Supplementary Figure 2. Decay rate of two-probe misclassified defectives estimated from Q4PCR data.

A) To test whether misclassified proviruses would decay similarly to nfl intact or defective proviruses, we used Q4PCR data to estimate the decay rate of proviral sequences which have a defect in packaging signal (PS) or HIV Env but are intact at the IPDA probe locations. Longitudinal data had n=35 time points for N=10 PWH. Dots are observations with dashed lines connecting individuals. Solid line is the best fit decay model and the shaded gray area is the 95% confidence interval on this estimate. B) Mean (dot) and 95% CI (vertical lines) indicate the decay rate of these misclassified proviruses in comparison to decay estimates for Q4PCR and IPDA intact and defective proviruses (see Fig 1). PS+Env defective proviruses that were intact at IPDA locations did not decay significantly (95% CI overlapping with zero) and their rates were similar to decay rates estimated for defective proviruses with Q4PCR and IPDA (i.e., teal and navy), suggesting misclassified proviruses might decay more similarly to nfl defective proviruses.

Supplementary References

1. Bacchetti, P. *et al.* Statistical analysis of single-copy assays when some observations are zero. *J Virus Erad* 5, 167–173 (2019).

2. Bruner, K. M. *et al.* A quantitative approach for measuring the reservoir of latent HIV-1 proviruses. *Nature* 566, 1–19 (2019).

3. Gandhi, R. T. *et al.* Selective Decay of Intact HIV-1 Proviral DNA on Antiretroviral Therapy. *J Infect Dis* 223, 225–233 (2021).

4. Antar, A. A. R. *et al.* Longitudinal study reveals HIV-1-infected CD4+ T cell dynamics during long-term antiretroviral therapy. *The Journal of clinical investigation* 2, 4629 (2020).

5. Falcinelli, S. D. *et al.* Longitudinal Dynamics of Intact HIV Proviral DNA and Outgrowth Virus Frequencies in a Cohort of Individuals Receiving Antiretroviral Therapy. *J Infect Dis* 224, 92–100 (2020).

6. Levy, C. N. *et al.* A highly multiplexed droplet digital PCR assay to measure the intact HIV-1 proviral reservoir. *Cell Reports Medicine* 100243 (2021) doi:10.1016/j.xcrm.2021.100243.

7. White, J. A. *et al.* Measuring the latent reservoir for HIV-1: Quantification bias in near full-length genome sequencing methods. *Plos Pathog* 18, e1010845 (2022).

8. Peluso, M. J. *et al.* Differential decay of intact and defective proviral DNA in HIV-1–infected individuals on suppressive antiretroviral therapy. *JCI Insight* 5, 23–14 (2020).
