# Supplementary material for: Impact of misclassified defective proviruses on HIV reservoir measurements
Source: Nat Commun. 2023 Jul 13;14:4186. doi: 10.1038/s41467-023-39837-z (PMC10345136; doi:10.1038/s41467-023-39837-z)
Supplement: Supplementary file 3 — Description of Additional Supplementary Files [file 41467_2023_39837_MOESM3_ESM.pdf]

File name: Supplementary Data 1

Description: Raw data comparison of Q4PCR and IPDA on the same samples, used for analysis and decay modeling. Measurements where intact HIV DNA was undetectable were set to half of the proviral frequency assuming 1 intact sequence in the number of cells[1] (see blue entries denoted by *). Samples having DNA quality or probe amplification issue by IPDA were excluded from all modeling analyses but included here for completeness (see red entries denoted by #).
