# Supplementary material for: Impact of misclassified defective proviruses on HIV reservoir measurements
Source: Nat Commun. 2023 Jul 13;14:4186. doi: 10.1038/s41467-023-39837-z (PMC10345136; doi:10.1038/s41467-023-39837-z)
Supplement: Supplementary file 5 — Reporting Summary [file 41467_2023_39837_MOESM5_ESM.pdf]

nature portfolio

Corresponding author(s): Daniel Reeves

Last updated by author(s): Feb 28, 2023

# Reporting Summary

Nature Portfolio wishes to improve the reproducibility of the work that we publish. This form provides structure for consistency and transparency in reporting. For further information on Nature Portfolio policies, see our Editorial Policies and the Editorial Policy Checklist.

## Statistics

For all statistical analyses, confirm that the following items are present in the figure legend, table legend, main text, or Methods section.

| n/a | Confirmed | |
|---|---|---|
| ☐ | ☒ | The exact sample size (*n*) for each experimental group/condition, given as a discrete number and unit of measurement |
| ☐ | ☒ | A statement on whether measurements were taken from distinct samples or whether the same sample was measured repeatedly |
| ☐ | ☒ | The statistical test(s) used AND whether they are one- or two-sided<br>*Only common tests should be described solely by name; describe more complex techniques in the Methods section.* |
| ☒ | ☐ | A description of all covariates tested |
| ☒ | ☐ | A description of any assumptions or corrections, such as tests of normality and adjustment for multiple comparisons |
| ☐ | ☒ | A full description of the statistical parameters including central tendency (e.g. means) or other basic estimates (e.g. regression coefficient) AND variation (e.g. standard deviation) or associated estimates of uncertainty (e.g. confidence intervals) |
| ☐ | ☒ | For null hypothesis testing, the test statistic (e.g. $F$, $t$, $r$) with confidence intervals, effect sizes, degrees of freedom and $P$ value noted<br>*Give P values as exact values whenever suitable.* |
| ☒ | ☐ | For Bayesian analysis, information on the choice of priors and Markov chain Monte Carlo settings |
| ☒ | ☐ | For hierarchical and complex designs, identification of the appropriate level for tests and full reporting of outcomes |
| ☐ | ☒ | Estimates of effect sizes (e.g. Cohen's *d*, Pearson's *r*), indicating how they were calculated |

*Our web collection on statistics for biologists contains articles on many of the points above.*

## Software and code

Policy information about availability of computer code

| | |
|---|---|
| Data collection | No software was used for data collection. |
| Data analysis | Data analysis code is publicly available and released at https://github.com/dbrvs/IPDAmodel/releases/tag/v1. |

For manuscripts utilizing custom algorithms or software that are central to the research but not yet described in published literature, software must be made available to editors and reviewers. We strongly encourage code deposition in a community repository (e.g. GitHub). See the Nature Portfolio guidelines for submitting code & software for further information.

## Data

Policy information about availability of data

All manuscripts must include a data availability statement. This statement should provide the following information, where applicable:

- Accession codes, unique identifiers, or web links for publicly available datasets
- A description of any restrictions on data availability
- For clinical datasets or third party data, please ensure that the statement adheres to our policy

A data availability statement is included in the manuscript including raw data in Supplementary Information and accession numbers at Genbank.

## Human research participants

Policy information about studies involving human research participants and Sex and Gender in Research.

| | |
|---|---|
| Reporting on sex and gender | Characteristics of study participants including sex and gender can be found in table S1 in https://doi.org/10.1073/pnas.2117630119. All study participants were of male sex based on self-reporting. No sex-based analyses were performed. |
| Population characteristics | 5 participants were White, 3 Black and 2 Unknown/Hispanic-Latino. Median age at last study time-point 42 years (range 27-61). 8 participants initiated antiretroviral therapy after chronic infection, 2 participants initiated antiretroviral therapy after early infection. |
| Recruitment | Study participants were enrolled at the Clinical Center, National Institute of Allergy and Infectious Diseases, National Institutes of Health, Bethesda, MD 20892, USA. |
| Ethics oversight | This study was approved by the institutional review board of the National Institute of Allergy and Infectious Diseases, NIH, and registered on ClinicalTrials.gov, NCT00039689. |

Note that full information on the approval of the study protocol must also be provided in the manuscript.

# Field-specific reporting

Please select the one below that is the best fit for your research. If you are not sure, read the appropriate sections before making your selection.

☒ Life sciences ☐ Behavioural & social sciences ☐ Ecological, evolutionary & environmental sciences

For a reference copy of the document with all sections, see nature.com/documents/nr-reporting-summary-flat.pdf

# Life sciences study design

All studies must disclose on these points even when the disclosure is negative.

| | |
|---|---|
| Sample size | Sample size was determined by study enrollment numbers and longitudinal time points were included to allow for sufficient modeling granularity. |
| Data exclusions | Excluded time points are noted in Supp Table 1. HIV reservoirs do not stabilize immediately after initiation of ART, so time-points <6 mo after starting ART were excluded. |
| Replication | All code is completely reproducible and available. |
| Randomization | No groups were created |
| Blinding | No blinding was required because no intervention was applied and because no covariates were studied across population groups. |

# Reporting for specific materials, systems and methods

We require information from authors about some types of materials, experimental systems and methods used in many studies. Here, indicate whether each material, system or method listed is relevant to your study. If you are not sure if a list item applies to your research, read the appropriate section before selecting a response.

### Materials & experimental systems

| n/a | Involved in the study | |
|---|---|---|
| ☒ | ☐ | Antibodies |
| ☒ | ☐ | Eukaryotic cell lines |
| ☒ | ☐ | Palaeontology and archaeology |
| ☒ | ☐ | Animals and other organisms |
| ☐ | ☒ | Clinical data |
| ☒ | ☐ | Dual use research of concern |

### Methods

| n/a | Involved in the study | |
|---|---|---|
| ☒ | ☐ | ChIP-seq |
| ☒ | ☐ | Flow cytometry |
| ☒ | ☐ | MRI-based neuroimaging |

# Clinical data

Policy information about clinical studies

All manuscripts should comply with the ICMJE guidelines for publication of clinical research and a completed CONSORT checklist must be included with all submissions.

| | |
|---|---|
| Clinical trial registration | NCT00039689 |
| Study protocol | ClinicalTrials.gov |
| Data collection | Data were collected at Rockefeller University NY in an ongoing observational study which was initiated in July 2002. |
| Outcomes | Clinical outcomes were not considered in the present study, which is a secondary natural history analysis of the original clinical trial. |
